# Supplementary material for: Are carbon emissions trading and green financial instruments synergistic? -Comprehensive quantitative research based on content analysis
Source: PLoS One. 2024 Mar 7;19(3):e0298601. doi: 10.1371/journal.pone.0298601 (PMC10919601; doi:10.1371/journal.pone.0298601)
Supplement: S1 Data — (ZIP) [file pone.0298601.s001.zip › Data/地级市Z_F工作报告.zip!/╣≤╓▌/╣≤╤⌠╩╨/└┤╫╘╣1⁄2╓┌║┼í╛┬φ┐╦╩2╛▌═°í┐_2018.docx]

各位代表：

　　现在，我代表市人民政府向大会作工作报告，请予审议，并请市政协委员和其他列席人员提出意见。

　　一、2017年政府工作回顾

　　过去一年，我们认真学习贯彻党的十九大精神和习近平总书记在贵州省代表团重要讲话精神，以习近平新时代中国特色社会主义思想为指导，自觉肩负起全省经济社会发展“火车头”、“发动机”的历史责任，切实增强政治意识、大局意识、核心意识、看齐意识，深入贯彻新发展理念，牢牢把握稳中求进工作总基调，守好发展和生态两条底线，以供给侧结构性改革为主线，大力实施大扶贫、大数据、大生态战略行动，全面小康社会建设取得新的进展，全面深化改革迈出坚实步伐，全面依法治市不断深入实施，全面从严治党持续纵深推进，圆满完成全年经济社会发展目标任务，为加快建设公平共享创新型中心城市奠定了坚实基础。

　　经济增长稳中有进。地区生产总值完成3537.96亿元，增长11.3%，经济总量在省会城市中上升1位，经济增速连续7年位居省会城市前2位，城市综合实力在中国百强城市中列第41位，上升9位。

　　经济结构优化调整。三次产业结构比重为4.2∶38.8∶57；高技术产业增加值142.91亿元，占规模以上工业增加值的比重为18.3%；预计非公有制经济总量占全市经济总量的55%，比上年提高2个百分点；固定资产投资3850.6亿元，增长18.1%；社会消费品零售总额1335.28亿元，增长11.7%；外贸进出口总额29.92亿美元，增长25.3%。

　　质量效益稳步提升。一般公共预算收入377.77亿元，增长8.01%；规模以上工业企业利润总额206.94亿元；规模以上工业产销率95.3%，比上年提高0.5个百分点；绿色经济占地区生产总值比重为39%，成为中国绿色发展优秀城市和全国质量强市示范城市。

　　发展动能显著增强。大数据企业主营业务收入800亿元，增长21%，软件和信息技术服务业业务收入145亿元，增长20%，网络零售交易额78.44亿元，增长45%，占全省比重分别超过50%、70%、59%；成交技术合同2947项，增长136%，对外输出技术合同金额43.98亿元，增长279%；万人发明专利拥有量11.19件，在西部省会城市中名列前茅，科技进步对经济增长的贡献率达到60%；全社会研发投入预计50亿元，占地区生产总值的1.42%。

　　生态质量持续向好。饮用水水源地水质达标率100%，地表水国控、省控断面水质优良率93%以上,南明河治理顺利实现除臭目标；新增城市公共绿地112万平方米，森林覆盖率48.66%；环境空气质量优良率95.1%，首次达到国家二级标准，在全国74个重点城市中列第9位。

　　改革红利不断释放。公立医院全面取消药品加成，实行零差率销售；466个村通过“三变”改革实现农民变股东9.22万人，带动1.67万农村低收入困难群众稳定增收；营改增政策性减税、政府性基金降费等为企业减负28.78亿元；全年新增市场主体12.3万户，新增注册资本5279亿元。

　　民生保障加快改善。新增城镇就业23.19万人，其中：农村劳动力转移就业3.82万人；城镇、农村常住居民人均可支配收入分别达到32186元、14264元，分别增长9.1%、10%；农村低收入困难群众人均保障标准提高到4800元/年，城市低保家庭人均保障标准提高到7500元/年；以更大的力度、更扎实的举措推进“菜篮子”工程建设，新建高标准蔬菜保供基地11600亩，蔬菜产量达到5000万公斤以上，蔬菜价格稳中有降，物价整体水平保持稳定。

　　一年来，我们迎难而上，砥砺前行，克难攻坚，开拓进取，建设公平共享创新型中心城市取得新的进展。

　　（一）深化供给侧结构性改革，供给结构加快改善。“三去一降一补”深入扎实推进。关停落后煤炭产能54万吨，商品房库存量保持在合理可控区间，“去”的效果明显。累计减少企业用电和税费成本37.08亿元，“降”的力度加大。市级财政对“三农”的投入增加到20.05亿元，增长4.8%，交通、教育、卫生等领域完成投资超过400亿元，“补”的步伐加快。工业结构转型升级不断加快。大力实施“千企引进”和“千企改造”工程，新开工工业项目157个，吉利汽车发动机、汉能移动新能源等项目开工建设。实施传统产业技术改造项目326个，完成投资183亿元，中铝“退城进园”项目一期建成投产。全市规模以上工业增加值完成817.8亿元，增长9.7%。农业产业结构调整持续深化。调整农业种植结构33.58万亩，新认证“三品一标”产品161个，农产品加工业总产值超过700亿元，规模以上企业农产品加工转化率达到45%。成功举办两届农业嘉年华，寻味贵州、心净桃源等一批农旅一体化项目精彩亮相，现代农业增加值占一产增加值比重提高到36%，农业和农村利益联结机制不断完善。现代服务业结构优化提升。平安银行贵阳分行开业，贵州金融城金融服务企业达到150个，金融业集聚发展效应开始显现。新增上市公司3个、新三板挂牌公司4个，便捷支付城市建设取得新进展。金融机构人民币存款、贷款余额双双突破万亿元，分别达到10814.51亿元和10403.12亿元。创建国家全域旅游示范区工作扎实推进，建成旅游厕所180个，荣获全国厕所革命先进市，新增旅游景区6个，青岩古镇成为国家5A级景区，填补了我市没有国家5A级景区的空白。全年接待游客1.49亿人次，实现旅游总收入1871.95亿元，分别增长34.1%、34.7%。市西滨河商业街开业运营。贵阳公路港城市物流中心投入运营，德国敦豪全球货运贵阳分公司成功落户，贵阳货车帮与江苏运满满战略重组圆满完成，成为全国最大的互联网货运企业。电子商务交易额突破1000亿元，增长33.1%。

　　（二）深入实施大扶贫战略行动，精准扶贫成效显著。脱贫攻坚“四场硬仗”强力推进。扎实开展脱贫攻坚“春季攻势”、“夏季大比武”和“秋季攻势”，全力以赴打好脱贫攻坚“四场硬仗”，建成农村“组组通”公路1165公里；易地扶贫搬迁519户2042人；实施产业扶贫项目139个，带动3.7万户11.2万人实现稳定增收；免除1.8万名困难家庭学生学杂费5963万元，19.3万农村学龄前儿童和中小学生吃上免费营养餐；“四重医疗保障”救助低收入人口2.8万多人。改造农村危房2061户，建设小康房2500套。大市场带动大扶贫扎实有效。贵阳北部农产品电商物流园开工建设，新增惠民生鲜超市50个，改造提升农贸市场66个，设立产业扶贫销售专区2万多平方米，生鲜农产品配送车辆达到100辆。实施“1+9”省内结对帮扶项目59个，带动省内其他市（州）13万贫困人口脱贫，在全省决战脱贫攻坚中不断作出贵阳贡献。

　　（三）深入实施大数据战略行动，创新动能持续增强。全力推进数字经济加速发展。以打造“中国数谷”为目标，突出抓好大数据“聚通用”，建成国家级互联网骨干直联点城市，互联网出省带宽达到6730Gbps，电信、移动、联通和多彩贵州广电云实现行政村光纤全覆盖。建成16个大数据产业聚集基地，数据中心服务器投运3.5万台。41个市属部门实施“数据铁笼”工程，筑民生、社会和云等社会管理服务APP上线运行。新增规模以上大数据企业51个，大数据企业纳税额110亿元，增长20%。大数据与实体经济加快融合。贵阳市成为国家产融合作试点城市，新云电子、同济堂成为国家智能制造综合标准化与新模式应用示范企业，贵州轮胎、詹阳动力等10个企业成为国家信息化和工业化融合管理体系贯标试点企业，航天电器、雅光电子等37个企业入选全省“大数据+工业”深度融合试点示范企业，大数据与实体经济深度融合指数达到42.5，比全省平均水平高8.7。大数据引领科技创新成果丰硕。成立全国首个大数据国家工程实验室，成为首个国家大数据及网络安全示范试点城市、健康医疗大数据中心国家试点城市、国家中小企业知识产权战略推进工程试点城市。政府数据共享开放总体解决方案成为国家推荐方案，《贵阳市政府数据共享开放条例》颁布施行，主导编制的贵州大数据地方标准发布实施。建成华为软件开发云创新中心、贵州伯克利大数据创新研究中心等8个创新平台，启动建设全国首个人工智能开放创新平台，新增省级以上科技企业孵化器8个，建成省级以上工程实验室、重点实验室、工程技术（研究）中心8个，引进高层次和紧缺大数据人才834人。

　　（四）深入实施大生态战略行动，生态建设强力推进。问题整改坚决有力。中央环保督察中受理的1681件群众信访投诉件全部办理完毕，24个集中式饮用水水源地保护区环境违法问题全部处理。生态修复效果明显。植树造林16.45万亩，山体治理6000多亩，7个市级示范公园建成开放。污染治理扎实有效。4个污水处理厂建成投用，污水日处理能力新增14.5万吨，花溪城市生活垃圾焚烧处理项目投入使用。完成市西河、贯城河等南明河支流治理，建成22条夜市规范摊区示范街。环境保护更加严格。全面实行政府、民间“双河长制”，全面划定永久基本农田。立案查处环境违法案件480起、林业违法案件574起，持续发出爽爽贵阳拒绝污染的最强音。

　　（五）深入实施新型城镇化战略，城乡面貌越变越美。城市规划水平不断提升。大力推进“多规融合”，划定城市开发边界，加强城市风貌管控，成为国家城市设计试点城市。贵阳市土地利用总体规划、城市总体规划修改后获得国家批准，编制完成乡镇、村庄建筑风貌导则，提升重点区域、特色村庄规划水平。基础设施短板加快补齐。交通基础设施建设大会战强力推进，贵遵高速公路复线建成通车，高速公路通车里程达到581公里，提等改造农村公路300公里。人民大道、旅游公路环线启动建设，城市轨道交通1号线局部试运营。建成骨干水源工程2个，年供水能力新增1679万立方米。发展农村耕地灌溉面积6万亩，10.42万农村人口饮水安全问题得到解决。城乡人居环境持续改善。完成棚户区城中村改造3.5万户、420万平方米，改造提升老旧小区院落200个，整治背街小巷215条。建设示范小城镇22个，建成美丽乡村提高型示范点57个，完成村庄环境综合整治138个，实施农村生活垃圾和生活污水处理示范项目33个，新建和改造村民组群众服务中心54个。

　　（六）大力深化改革开放，动力活力有效迸发。深化改革不断推进。落实国家和省级改革试点任务67项，完成144项年度改革任务，形成了一批在全国有影响的改革成果。成立统筹城乡“三变”改革专门机构，32个城市“三变”试点项目扎实推进，53.2%的行政村进行了农村“三变”改革。“放管服”改革深入推进，全面取消非行政许可类审批，下放行政审批事项30项，市级行政审批时限在法定时限基础上压缩60%以上，取消行政审批中介服务事项7项、涉企涉民证明事项42项，以政府权力的“减法”换取市场活力的“加法”。市属国有企业战略性重组强力推进，编制完成市属国有企业战略性重组方案，深化国有企业改革迈出坚实步伐。扩大开放取得突破。建设内陆开放型经济试验区行动计划加快推进，贵阳国家经济技术开发区成为国家知识产权试点园区，贵阳国家高新技术产业开发区成为全国双创示范基地，贵州双龙航空港经济区成为国家级临空经济示范区和国检试验区，贵阳综合保税区成为全省外贸转型升级示范基地。积极融入“一带一路”、长江经济带等国家战略，深化京筑、泛珠三角区域合作，成功举办2017中国国际大数据产业博览会、2017生态文明试验区贵阳国际研讨会等重大活动。引进国内外500强企业11个，总数达到98个，引进省外到位资金1638.5亿元，增长16%，实际直接利用外资13.45亿美元，增长20.1%。

　　（七）全力保障和改善民生，人民生活水平加快提升。十件民生实事顺利完成，全年民生领域支出376亿元，占一般公共预算支出的65%。创建充分就业城市全面启动。在全国率先开展创建充分就业城市工作，充分就业社区和充分就业行政村创建率分别达到85%和78%。教育立市战略深入实施。新建和改扩建中小学、幼儿园294所，新增学位3.12万个。贵阳学院成为硕士学位授予单位，贵阳职业技术学院、贵阳护理职业学院、贵阳幼儿师范高等专科学校顺利通过省级优质高等职业学校评估。精神文明建设成果丰硕。国家公共文化服务体系示范区创建成果持续巩固，建成县级图书馆2个、文化馆1个，连续三次荣膺全国文明城市称号。健康贵阳建设步伐加快。建成健康细胞工程示范点20个，乡（镇）中心卫生院全部达到甲等标准，乡级以上公办医疗机构实现远程医疗全覆盖。全民健身运动广泛开展，建成村级全民健身工程项目60个，成功举办中超联赛、贵阳国际马拉松赛、全国田径冠军大奖赛等重大赛事，健康城市建设初见成效。平安贵阳建设成效显著。深入开展“两严一降”和禁毒人民战争，安全生产事故发生起数和死亡人数持续下降，人民群众安全感上升到98.43%，创十年来最高记录。拥军爱军工作走在全国前列，公共法律服务体系不断完善，社会治理创新能力迈上新台阶。

　　国家安全、国防动员、民族宗教、人防、档案、侨务、气象、地方志、双拥优抚、工青妇、红十字、老龄和残疾人等事业全面发展，人民群众获得感、幸福感、安全感和对发展的满意度持续上升。

　　（八）全面推进政府自身建设，行政效能有效提升。自觉同以习近平同志为核心的党中央保持高度一致，认真学习宣传贯彻党的十九大精神和习近平总书记在贵州省代表团重要讲话精神，扎实推进“两学一做”学习教育常态化制度化，深入开展大学习、大宣讲、大调研、大宣传、大落实活动。大力推动政府系统全面从严治党，严格遵守和执行廉洁自律各项规定，持续纠“四风”、转作风，不断强化行政监察和审计监督，深入开展民生监督，狠抓正风肃纪，全年立案1721件，党纪政纪处分1582人、移送司法机关45人。法治政府建设不断推进，提请市人大常委会审议地方性法规草案4件，制定政府规章9件，全面清理政府规章158件。办理人大代表建议254件、政协委员提案411件，办复率均达到100%。

　　各位代表，过去的一年，发展成绩来之不易、发展成就振奋人心、发展经验弥足珍贵。这是省委、省政府和市委坚强领导、科学决策的结果，是市人大、市政协监督支持、群策群力的结果，更是全市各族人民齐心协力、拼搏奋斗的结果。我代表市人民政府，向全市各族人民致以崇高的敬意！向人大代表、政协委员，向各民主党派、工商联、人民团体、无党派人士，向驻筑人民解放军、武警官兵和人民警察，向中央、省驻筑单位和兄弟市（州），向贵阳市的老领导、老同志以及所有关心支持贵阳发展的各界人士表示衷心的感谢！

　　在看到成绩的同时，我们清醒认识到，贵阳虽然是省会城市，但发展不平衡不充分的问题还比较突出，经济社会发展面临诸多的困难和挑战。主要是：经济实力不强。经济总量在省会城市中偏小，作为全省经济社会发展的“火车头”和“发动机”，牵引力不大、带动力不强。产业层次不高。传统产业比重大，新兴产业规模小，大数据与实体经济的融合有待提升。投资结构不优。产业投资总量少，民间投资比重低，经济稳定持续发展压力大。创新能力不够。科技研发投入不足，企业创新主体作用发挥不够，创新型人才体系不健全，科技成果转化率低。风险隐患不少。金融领域、社会领域、安全生产领域、生态环境领域的风险隐患还没有得到有效遏制，维护社会稳定任务繁重。服务能力不足。有的地方和部门担当作为不够，少数干部研究问题、破解难题能力不强，不作为、不会为、乱作为的现象不同程度存在。我们要进一步增强省会意识和危机意识，迎难而上、锐意进取，坚决有效地解决好突出问题，全力做好政府工作，让省委、省政府和市委放心，让人民群众满意。

　　二、2018年工作安排

　　今年是贯彻党的十九大精神的开局之年，是改革开放40周年，是决战脱贫攻坚、决胜全面小康和实施“十三五”规划承上启下的关键一年。政府工作要适应新时代、聚焦新目标、落实新部署，为决战脱贫攻坚、决胜全面建成小康打下坚实基础。中国特色社会主义进入新时代，推动高质量发展，国家实施创新驱动、乡村振兴、军民融合等重大战略，贵州建设三大国家试验区都给我们带来重大机遇；守好发展和生态两条底线、转变经济发展方式、优化经济结构、转换生产动能、跨越高速增长阶段转向高质量发展阶段重要关口是我们面临的重大挑战和考验。机遇与挑战并存，我们要紧紧抓住机遇，冷静应对挑战。

　　紧紧抓住机遇，我们必须快马加鞭。“一万年太久，只争朝夕。”我们要不忘初心、牢记使命，以更加振奋的精神，更加昂扬的斗志，拥抱新时代、聚焦新目标、展现新气象、实现新作为。要有“说办就办，马上就办”的精神，一分也不能耽误，一秒也不能停滞，把时间这个最稀缺的资源利用好、发挥好。紧紧抓住机遇，我们必须苦干实干。空谈误国、实干兴邦。我们要不忘初心、牢记使命，在解放思想上有新进步、在改革开放上有新突破、在后发赶超上有新成效、在全面建成小康社会进程中有新跨越。紧紧抓住机遇，我们必须善作善成。炼就真本领、下足硬功夫。我们要不忘初心、牢记使命，奋力提升“八大本领”，大力培育专业精神，敢做敢为，善谋善断。在干中突破、在干中跨越、在干中建成更高水平全面小康。

　　冷静应对挑战，我们必须坚定信心。不畏浮云遮望眼，风物长宜放眼量。我们要牢记嘱托、感恩奋进，把握大局大势，保持清醒头脑，不因成绩而骄傲，不因困难而退缩，始终保持战略定力和发展耐力，坚定必胜信心，奋勇闯关夺隘。冷静应对挑战，我们必须勇于改革。长风破浪会有时，直挂云帆济沧海。我们要牢记嘱托、感恩奋进，用好改革关键一招，始终突出问题导向，精准实施靶向治疗，化挑战为机遇。冷静应对挑战，我们必须奋力创新。惟创新者强，惟创新者胜。我们要牢记嘱托、感恩奋进，大力弘扬新时代贵州精神，下好创新先手棋，厚植发展新优势，开辟新天地，开创新未来，推动我们的事业不断从胜利走向胜利。

　　今年政府工作的总体要求是：全面贯彻落实党的十九大精神、习近平总书记在贵州省代表团重要讲话精神和中央经济工作会议精神，以习近平新时代中国特色社会主义思想为指导，加强党对一切工作的集中统一领导。按照中央、省委和市委的决策部署，坚持新发展理念，坚持稳中求进工作总基调，紧扣经济社会主要矛盾的变化，按照高质量发展的要求，统筹推进“五位一体”总体布局，协调推进“四个全面”战略布局。牢记嘱托、感恩奋进，大力培育和弘扬新时代贵州精神，坚持以供给侧结构性改革为主线，坚持主基调主战略，统筹稳增长、促改革、调结构、惠民生、防风险，全力推动经济发展质量变革、效率变革、动力变革，加快构建具有贵阳特色的现代化经济体系。坚决打好防范化解重大风险、精准脱贫、污染防治攻坚战，守好发展和生态两条底线，加强和改善民生，以城乡“三变”改革为基本路径，高一格快一步深一层实施大扶贫、大数据、大生态三大战略行动，促进全市经济社会持续健康发展，着力打造以生态为特色的世界旅游名城，加快建设公平共享创新型中心城市，奋力开创百姓富生态美的爽爽贵阳新未来。

　　今年经济社会发展主要预期目标是：地区生产总值增长11%左右，规模以上工业增加值增长10%，固定资产投资增长19%，社会消费品零售总额增长11.5%，外贸进出口总额增长13%，一般公共预算收入增长8%，城镇、农村常住居民人均可支配收入分别增长9.5%和10%，金融机构人民币存款、贷款余额分别增长13%和14%；工业经济、民营经济、绿色经济、新经济占地区生产总值比重分别提高到24%、58%、42%和21%；城镇登记失业率控制在4.2%以内；蔬菜价格水平与周边省会城市持平，居民消费价格涨幅控制在省调控目标范围内；单位地区生产总值综合能耗下降率、单位地区生产总值二氧化碳排放降低率控制在省下达目标范围内。

　　做好今年经济社会发展工作，要坚定不移地贯彻落实稳中求进工作总基调，正确处理经济发展中稳和进的关系，统筹兼顾、突出重点，坚持质量第一、效益优先，把高质量发展的根本要求体现到经济社会发展的各方面和全过程，在质的提升中实现量的增长，全力以赴抓好九项重点工作。

　　（一）紧紧围绕建成更高水平全面小康社会，全力以赴打好三大攻坚战。以“等不起”的紧迫感、“慢不得”的危机感、“坐不住”的责任感，坚决打好防范化解重大风险、精准脱贫、污染防治攻坚战，在全省率先建成更高水平全面小康社会、率先开启社会主义现代化新征程。一是坚决打好防范化解重大风险攻坚战。坚持主动防范、系统应对、标本兼治、守住底线，有效防范化解各种风险。严防和化解地方政府债务风险。深入推进预算绩效改革，严格执行政府性债务管理“七严禁”规定，建立政府性债务终身追责和责任倒查机制，“修明渠、堵暗道”，有序化解政府存量债务，严控隐性政府性债务增量。严防和化解金融风险。加大对网络借贷、众筹、融资担保等准金融业态的监管力度，严厉打击非法集资和传销，牢牢守住不发生区域性系统性金融风险的底线。严防和化解安全生产和社会稳定风险。进一步加强重点行业、重点领域安全生产隐患常态化排查整治，严格落实重大项目、重大政策社会稳定风险评估机制。做好土地房屋征收、环境保护、社会管理等方面的矛盾纠纷排查化解，加强自然灾害、公共卫生、食品药品安全、网络舆情等方面的风险防控和应急处置，坚决遏制重特大事故和群体性事件的发生。二是坚决打好精准脱贫攻坚战。尽锐出战、精准施策，高一格快一步深一层推进大扶贫战略行动。精准聚焦脱贫攻坚对象。大力推进20个特别困难村定点包干和100个低收入困难村结对帮扶，确保纳入全国扶贫开发信息系统的非兜底保障贫困人口全部从系统内退出。精准聚焦脱贫攻坚目标。确保农村低收入困难群体人均保障标准提高到6000元，调整农业种植结构58万亩，带动7.9万农村低收入困难群众实现解困。精准聚焦脱贫攻坚举措。深入推进农村“组组通”公路大决战，建成通组公路2570公里，30户以上的村民组全部通硬化路。完成新增2500户10048人易地扶贫搬迁房屋主体工程建设。加大教育医疗住房“三保障”力度，实现农村建档立卡困难家庭学生“两助三免”政策全覆盖，农村建档立卡困难家庭和低收入困难群众100%列入健康扶贫对象，普通门诊、普通住院、大病保险报销比例提高10%以上。改造农村危房760户，在全省率先全面消除农村危房。精准聚焦脱贫攻坚帮扶。加强冷链物流体系建设，新建惠民生鲜超市77个，建成贵阳北部农产品电商物流园，为省内其他市（州）贫困人口提供15万个就业岗位，带动省内结对帮扶贫困县10万贫困人口实现脱贫，脱贫人口人均年收入达到5000元以上。三是坚决打好污染防治攻坚战。坚持生态优先、绿色发展，统筹山水林田湖草系统治理，加强生物多样性保护，加快构建“一河百山千园”自然生态体系。筑牢绿色屏障。植树造林80万亩，山体治理3400亩；新增城市公共绿地100万平方米以上，森林覆盖率提高到52%，环境空气质量优良率保持在90%以上。守护绿色家园。确保按时完成中央环保督察反馈问题整改。编制完成中心城区城市排水系统总体规划，以南明河治理为重点，因地制宜建设一批大、中、小、微型污水处理设施，建成污水处理厂7个，完成19条城区排水大沟清污分流整治，确保南明河实现变清目标。开展生活垃圾分类工作，城市生活垃圾无害化处理率达到97.8%。大力推进农村人居环境提升行动，全市农村生活垃圾治理覆盖率、污水治理覆盖率均达到70%以上。发展绿色经济。实施绿色经济倍增计划和绿色制造三年专项行动，推进20个传统制造业绿色化改造和绿色制造技术创新运用重大项目建设，绿色经济占地区生产总值比重达到42%。完善绿色制度。健全自然资源资产有偿使用和生态补偿制度，推进自然资源统一确权登记。全面推行生态环境损害赔偿制度，启动资源环境承载力评价，推进环境污染第三方治理，探索建立环境污染跨区域联防联治机制。强化生态环保“两把利剑”、“两个问责”的作用，实现环保督察巡查全覆盖。

　　（二）紧紧围绕供给侧结构性改革，全力以赴做强实体经济。深入推进供给侧结构性改革，把提高供给体系质量作为主攻方向，继续做好“三去一降一补”，着力化解过剩产能，推动市场出清。深入实施创新驱动、质量强市发展战略，加快构建具有贵阳特色的现代化产业体系，推动产业发展向中高端水平迈进。着力推进工业智能化发展。深入实施工业发展倍增行动计划，着力打造军民融合、汽车制造、铝及铝加工、医药食品、磷煤化工五个千亿级产业集群。加大“千企改造”和“千企引进”力度，实施一批工业机器人应用、数字化车间和智能工厂示范项目，改造升级传统企业300个，推动黔轮胎完成贵阳基地智能化整体改造提升，开工建设越南基地一期项目；引进一批技术含量高、成长性好、引领性强的优强企业拓展产业链，加快启动中铝“退城进园”二期建设，确保吉利汽车30万辆整车项目建成投产、宝能汽车制造项目主体工程完工，力争建成汉能移动新能源项目。全年实施重点工业项目120个，新增规模以上工业企业100个；完成工业投资750亿元，增长25%。着力推进服务业高端化发展。持续实施“引金入筑”工程，推动贵州金融城和金融北城联动发展，金融业增加值增长13%以上。实施新一轮大健康产业发展六项行动计划，力争大健康产业增加值增长20%。实施商贸服务业提升行动，强化中心城区商贸服务业龙头引领作用，推动线上线下融合创新和传统商圈提档升级、新兴商圈发展壮大，加快观山湖区现代服务产业试验区和清镇国家级示范物流园区建设。全年新增规模以上服务业企业100个、限额以上批发零售餐饮业企业100个、限额以上住宿业企业50个，现代服务业增加值占服务业增加值比重保持在52%以上。着力推进科技协同化创新。新增省级工程技术研究中心4个、省级企业技术中心3个，新认定高新技术企业150个以上，力争全社会研发投入占地区生产总值比重提高到1.8%，科技进步对经济增长的贡献率提高到61%。着力推进质量品牌化建设。大力弘扬“工匠”精神，激发和保护企业家精神，巩固提升全国质量强市示范城市创建成果。开展质量提升贵阳行动，加强品牌建设，建立完善产品标准体系，新增全国知名品牌示范区1个，培育标准领跑者企业30个。

　　（三）紧紧围绕打造中国数谷，全力以赴发展数字经济。加快推进国家大数据综合试验区核心区建设，保持大数据先行优势，把大数据打造成为引领经济社会高质量发展的强大引擎。加快推动大数据产业创新发展。建好用好国家大数据工程实验室等创新平台，高水平、高速度规划建设百花数谷，推进中国南方数据中心示范基地、中国人工智能开放创新平台、健康医疗大数据中心国家试点城市建设。加快贵阳国际软件产业园、贵州阿里巴巴产业基地等10个大数据产业园建设，打造两个50亿元级大数据产业基地。新增规模以上大数据企业50个，全市大数据企业主营业务收入突破1000亿元，增长25%；软件和信息技术服务业业务收入174亿元，增长20%；规模以上电子信息制造业增加值27亿元，增长15%；网络零售额102亿元，增长30%。进一步完善人才引进机制，完成大数据“十百千万”人才培养计划，形成多层次、多类型的大数据人才队伍。加快推进大数据与实体经济融合发展。扎实开展“千企融合”行动，打造大数据应用场景100个以上，实施工业、农业、金融、物流等领域的大数据融合示范项目30个，全市重点工业企业数字化研发设计普及率超过70%，大数据与实体经济深度融合指数达到44以上。加快推进大数据提升政府治理能力。抓好政府数据“聚通用”，加强政府数据开放共享体系建设，进一步推动政务数据一体化，全市政务应用系统100%接入“云上贵州”贵阳分平台、政府数据100%实现共享交换。开展基于区块链技术的治理科技应用示范，推进智慧市场监管、智慧城管、智慧信用等政府治理大数据应用。加快完善社会征信体系，建成国家企业信用信息公示系统贵阳分中心。加快推进大数据保障和改善民生。建设智慧教育大数据融合分析平台、智能化交通出行大数据平台。拓展筑民生平台的服务功能，开展30个典型示范应用，提高民生服务的精准化、公平化、共享化水平。加快推进大数据安全保障能力提升。全力推进国家大数据安全靶场建设，完善大数据网络安全监管服务平台。强化网络与信息安全通报机制，全面开展关键基础设施和重大信息系统等级保护工作。打造大数据及网络安全示范基地，构筑具有贵阳特色的大数据及网络安全产业体系和保障体系。

　　（四）紧紧围绕建设现代城市，全力以赴完善城市功能。启动新一轮土地利用总体规划、城市总体规划编制工作，拓展、优化城市空间布局，完善城市功能配套，提升城市公共服务水平，建设具有贵阳特色的现代化城市，持续增强省会城市的辐射力和带动力。突出抓好交通基础设施建设。加快构建“五环十八射”路网格局，启动建设贵阳综合保税区至开阳产业大道、三马片区“四横四纵”骨架路网，建成人民大道北段、花冠路、旅游公路环线示范段、同城大道黔灵山路至扎佐段、新东二环，开工建设高速公路160公里。建成高铁贵阳东站，开工建设轨道交通3号线一期、确保1号线全线试运营。加快推进城市综合管廊、海绵城市建设。强力推进棚户区城中村改造。高标准、高速度、高效率、高质量推进人民大道沿线、花冠路沿线以及三马片区、盐务街片区、六广门片区等棚户区城中村改造，实施棚户区城中村改造5.8万套、950万平方米，惠及20.3万人。加快推动区域融合发展。加快推进三县一市和中心城区、中心城区和中心城区之间的融合发展，加快推动贵阳与贵安新区同城化发展，不断拓展城市发展空间。着力提升城市精细化管理水平。建立健全城市精细化治理体系，大力实施占道经营整治、违法违章建设整治、市政基础设施综合整治、绿化文明施工作业管理等十大专项行动，鼓励和引导市民积极参与城市管理，形成共建共治共享的良好城市发展格局。

　　（五）紧紧围绕乡村振兴战略，全力以赴夯实“三农”基础。坚持新型城镇化和乡村振兴两手抓，坚持农业农村优先发展，以产业兴旺为重点、生态宜居为关键、乡风文明为保障、治理有效为基础、生活富裕为根本，协调推进农村经济建设、政治建设、文化建设、社会建设、生态文明建设和党的建设，促进乡村全面发展。在完善农村基础设施上更加用力。开工建设乡镇与高速公路互通联络线100公里，按照“四好农村路”标准提等改造农村公路500公里。加快孟关等3个供水厂建设，抓好黔中水利枢纽工程配套项目建设，新建农村小型水利灌溉工程79个，开展农村消防水源点建设。加大农村电网改造和管护力度，改善提升农村通讯条件，20户以上自然村光纤和4G网络覆盖率达到85%以上。在发展现代农业上更加用力。深入实施绿色农产品“泉涌”工程，以现代高效农业园区建设和农业嘉年华活动为载体，发展观光农业、体验农业，打造一批农旅一体、文旅一体精品项目，推动农业接二连三融合发展。突出农业绿色化、优质化、特色化、品牌化，新认证“三品一标”产品150个，启动生态原产地产品保护示范区申报工作，评定生态原产地产品10个以上。新增省级现代高效农业示范园区4个，引进培育农业龙头企业20个、农产品精深加工企业15个，农产品加工总产值达到770亿元以上。在促进城乡融合发展上更加用力。坚持以工补农、以城带乡，推动形成工农互促、城乡互补、全面融合、共同繁荣的新型工农城乡关系。启动建设特色小镇试点，围绕旅游公路环线规划建设一批产业型、资源型、生活型特色小镇。大力建设富美乡村，打造田园综合体30个，建成富美乡村示范村30个以上。在深化农村改革上更加用力。巩固和完善农村基本经营制度，以完善产权制度和要素市场化配置为重点，深入推进农村“三变”改革、农村金融改革、农村治理方式变革。新发展农民专业合作社50个以上、家庭农场30个以上，激活主体、激活要素、激活市场，让农业成为有奔头的产业，让农民成为有吸引力的职业，让农村成为安居乐业的美丽家园。

　　（六）紧紧围绕打造世界旅游名城，全力以赴培育城市品牌。以建设国家全域旅游示范区为抓手，推动旅游业由高速增长向优质发展转变，加快将贵阳打造成为以生态为特色的世界旅游名城。高质量打造精品旅游景区。大力实施旅游景区精品提升行动，加快恒大童世界、东方科幻谷、中国阳明文化园等核心吸引物建设，力争将天河潭创建成为国家5A级旅游景区，新增4A级旅游景区3个以上。高标准完善旅游功能配套。实施旅游服务标准化建设，实现重点旅游景区、城市主干道旅游国际化标识标牌全覆盖，实现4A级以上旅游景区免费WiFi、智能导游和电子讲解全配套。提升旅游服务功能，新增度假精品酒店、特色客栈10个以上，建设旅游厕所100个以上。高层次推动旅游业态创新。建成特色旅游文化街区3条、康养旅游项目2个、农旅一体化项目1个，打造旅游商品购物基地5个以上。全年接待游客人次、实现旅游总收入分别增长25%和30%，其中：入境游达到38万人次，推动旅游业持续“井喷”发展。高水平做好旅游宣传推介。实施入境旅游市场开拓三年行动计划，推出5条以贵阳为中心的国际精品旅游线路，让“多彩贵州•爽爽贵阳”风行天下。

　　（七）紧紧围绕重大项目建设，全力以赴扩大有效投资。把抓项目扩投资作为高质量发展的重要支撑，通过有效投资优化经济结构、保障改善民生、提高发展质量。突出抓好项目储备，增强发展后劲。把握国家政策导向，紧盯重点产业、重点行业、重点领域，精心做好项目谋划工作，做深做实前期工作，确保市级重大项目库投资总额动态保持在3万亿元以上，其中：产业类项目储备超过50%，年度可实施项目总投资超过1万亿元。突出抓好招商引资，做优投资结构。深入实施大招商行动计划，围绕发展实体经济开展精准招商，全年引进项目630个，其中：工业项目比重达到30%以上；引进国内外500强企业14个，引进到位资金突破2000亿元，增长25%；市级重点招商项目履约率达到95%以上，实际直接利用外资15.5亿美元，增长16%。突出抓好项目实施，做大投资总量。确保全年新开工重大工程和重点项目170个以上，纳入省重大工程和重点项目450个以上，完成投资1550亿元以上，力争民间投资比重达到50%以上。突出抓好改革创新，保障资金需求。在交通基础设施、水利设施、生态建设等领域推出一批PPP示范项目，最大限度吸引社会资本参与项目建设。继续深化政银企合作，积极争取国家和省专项建设基金、专项企业债券政策支持，确保项目建设资金需求。

　　（八）紧紧围绕激发动力活力，全力以赴深化改革开放。以开放促改革，以开放促发展，着力打造贵州内陆开放型经济试验区核心区和引领区。全面扩大对外开放。启动贵阳改貌铁路口岸申建工作，建成清镇普洛斯铁路港。加快设立贵阳国际邮件互换局，大力推进贵阳龙洞堡国际机场三期建设，开通2至3条洲际航班，新增1至2条国际及地区航线，贵阳龙洞堡国际机场旅客吞吐量突破2000万人次。加快瑞士（贵州）产业示范园区、粤桂黔高铁经济带合作试验区贵州园建设，着力推进贵阳国家经济技术开发区、贵阳国家高新技术产业开发区、贵州双龙航空港经济区、贵阳综合保税区提档升级，建成贵州（双龙）国检试验区。充分发挥好贵阳综合保税区功能，落实好出口共享激励机制，大力发展加工贸易、服务贸易、跨境电商，加快推进离岸服务外包，积极申报国家跨境电子商务综合试验区。精心筹办好2018中国国际大数据产业博览会、生态文明贵阳国际论坛2018年年会，持续发出贵阳好声音。全面推进城乡“三变”改革。完善农村土地、林业等资源入股机制，实施农村“三变”改革共享项目200个。以棚户区城中村改造为重点，全面推进城市“三变”改革共享试点项目32个，确保实现乡镇、社区“三变”改革全覆盖，惠及群众20万人以上。全面推进经济结构战略性转型。以旅游公路环线为平台，大力推进田园综合体、旅游综合体、康养旅游区、体旅休闲基地建设，着力发展生态、度假、养老、健康等绿色健康产业，推动农村经济结构加快向生态化、市场化、融合化转型。以人民大道、花冠路等为载体，大力推动棚户区城中村改造，发展楼宇经济、商圈经济，推动城市经济结构加快向高端化、现代化、服务化转型。以国有企业战略性重组为突破，深化国有企业改革，全面完成市属国有企业战略性重组任务，实现国资国企统一监管，推进国有企业做大做强做实，增强国有经济活力、控制力和影响力。全面深化“放管服”改革。大力优化政务服务环境，实施市场准入负面清单制度，改善营商环境。严格实行项目并联审批，全面推行“双随机、一公开”制度。扎实开展“减证便民”专项行动，推进相对集中行政许可权、“多证合一”、“三集中三到位”等改革，大力推行和积极探索“网上办、一次办、同城办、邮政办”、“一网通办”、“一趟不跑”等便民利民新模式，建成贵阳市12315智能服务平台。

　　（九）紧紧围绕美好生活需要，全力以赴增进民生福祉。坚持以人民为中心的发展思想，解决好老百姓最急最忧最怨的问题,在幼有所育、学有所教、劳有所得、病有所医、老有所养、住有所居、弱有所扶上用情用力。大力实施就业创业提升工程、居民收入提升工程、人居环境提升工程、交通出行提升工程、教育供给提升工程、医疗健康提升工程、养老敬老提升工程、城市文明提升工程、智慧治理提升工程和平安建设提升工程等“十大民生提升工程”，让人民群众有更多的获得感、幸福感、安全感。

　　让人民生活更加美好幸福。加快创建充分就业城市，稳步推进创业孵化基地建设，深入开展大学生创业引领计划，大力推动农民工返乡创业，确保全年城镇新增就业23万人，其中:农村劳动力转移就业3.76万人。加快构建共同富裕的体制机制，切实增加人民群众工资性、经营性、财产性收入，确保城镇、农村常住居民人均可支配收入稳步增长，实现居民收入增长和经济发展同步、劳动报酬增长和劳动生产率提高同步。大力推进公交优先，对30个交通堵点进行常态化精准治理，新增停车位1万个，出台小客车环城高速公路免费行驶优惠政策，优化调整机动车牌照摇号政策及限行措施。

　　让人居环境更加舒适美丽。完成城镇规划区外村庄规划编制工作，实现村庄规划全覆盖。新建和改造提升公厕200个，建成人行过街设施30个，改造老旧小区院落150个，实施背街小巷综合整治200公里。完成全市使用15年以上旧电梯安全评估工作。公共图书馆藏书人均占有量达到1册以上，经常参加体育锻炼人数比例达到37%；巩固全国文明城市创建成果，多措并举提升市民文明素质，使城市让生活更美好、农村让城市更向往逐步成为现实。

　　让公共服务更加优质均衡。实施第三期学前教育行动计划，开展义务教育优质均衡试点工作，全面推行义务教育学校管理标准，推进城乡教育一体化发展；实施中考、高考制度改革，推进新时代教师队伍建设改革，加快建设教育督导和质量监测体系；扎实推进“百校攻坚”行动计划，新建改扩建中小学、幼儿园50所，新增学位3万个，全面改造薄弱学校10所，建成山村幼儿园30所；推进贵州清镇职教城提升和孔学堂三期项目建设。加快建设全国健康城市，深入实施“百院攻坚”行动计划，全面完成基层医疗卫生服务能力提升行动计划，建设各类医院13个，全市社区卫生服务中心标准化建设达标率80%以上，建成健康细胞工程示范点30个以上，蝉联国家卫生城市；积极创建全国全民运动健身模范城市，广泛开展全民健身运动。推进养老事业发展，力争建设社区老年人日间照料中心20个以上、农村幸福养老院30个以上，每千名老年人拥有养老床位数达到35张。强化惠民便民服务，累计建成公益性惠民生鲜超市150个以上，实现城市行政社区全覆盖，打造15分钟便民服务生活圈；新建村民组群众服务中心70个，进一步拓展12345热线综合平台服务范围，更好地满足人民群众公共服务诉求。

　　让社会大局更加稳定和谐。提升社会治理社会化、法治化、专业化、智能化水平，建设新型智慧城市。实施停车场资源共享利用、物联网应用、数智共享社区等15个应用示范项目。把扫黑除恶专项斗争作为一项重大政治任务，摆到工作全局突出位置，列入重要议事日程扎实开展，保障人民安居乐业、社会安定有序、国家长治久安，进一步巩固党的执政基础；以创建全国禁毒工作示范城市为抓手，深入推进“两严一降”和禁毒人民战争，建设全域覆盖的人像大数据体系，进一步完善立体治安防控体系。实施食品安全战略，以打造智慧食品药品监管体系为抓手，大力开展“双安双创”，让人民群众吃得更放心。进一步加强安全生产、应急管理等工作，人民群众安全感保持在95%以上。加快公共法律服务平台建设，加大社会矛盾预防和化解力度，让社会更加安定有序，让百姓更加安居乐业。

　　支持军队、武警部队建设和监察委、法院、检察院工作。进一步加强国家安全、国防动员、民族宗教、人防、档案、侨务、气象、地方志、双拥优抚、工青妇、红十字、老龄和残疾人等工作，推动各项社会事业全面繁荣发展。

　　三、全面加强政府自身建设

　　新时代呼唤新作为，新时代要有新担当。我们要不忘初心、牢记使命，不驰于空想、不骛于虚声，进一步增强政府执行力落实力，建设党放心信任、人民满意信赖的政府，以实干实绩书写新时代贵阳答卷。

　　（一）坚持党的领导，把牢政治方向。旗帜鲜明讲政治，坚持“三个一以贯之”，树牢“四个意识”，增强“四个自信”，坚决维护以习近平同志为核心的党中央权威和集中统一领导，始终在政治立场、政治方向、政治原则、政治道路上同以习近平同志为核心的党中央保持高度一致。推进“两学一做”学习教育常态化制度化，扎实开展“不忘初心、牢记使命”主题教育，坚定理想信念，对党绝对忠诚，坚定不移把中央、省委和市委决策部署落到实处，确保政令畅通、令行禁止，维护好贵阳山清水秀的政治生态。

　　（二）强化依法行政，维护公平正义。坚持全面依法治国基本方略，大力实施依法治市战略，加快建设法治政府。推进依法行政，严格规范公正文明执法，完善社会共商共建共治共享机制，推进城市治理能力和治理体系现代化。完善重大行政决策程序制度、合法性审查制度、终身责任追究制度和责任倒查机制。依法接受市人大及其常委会的监督，自觉接受市政协的民主监督，主动接受社会和舆论监督，确保政府科学决策、民主决策。

　　（三）强化宗旨意识，永葆为民情怀。坚持以人民为中心的发展思想，大力建设人民满意的政府。把造福人民作为最大政绩，时时刻刻把老百姓的操心事烦心事扛在肩上、放在心上、抓在手上，一件一件办好办实，在改革发展中补齐民生短板，在高质量发展中增进民生福祉，不断提高人民群众的获得感、幸福感、安全感，让人民群众生活得更加幸福美满。

　　（四）强化责任担当，敢于攻坚克难。始终把党和人民的事业放在首要位置，坚持“干”字当头、“实”字为要，不断提升“八大本领”，大力推进“五步工作法”，以逢山开路、遇水架桥的勇气，改革创新、锐意进取，勇挑最重的担子、敢啃最硬的骨头、善接最烫手的山芋，以踏石留印、抓铁有痕的韧劲，苦干实干加油干，奋力谱写好新时代爽爽贵阳发展新篇章。

　　（五）强化勤政廉政，守牢廉洁底线。以永远在路上的执着把全面从严治党引向深入。大兴调查研究之风、真抓实干之风、勤俭节约之风，驰而不息纠正“四风”、转变作风，力戒形式主义、官僚主义。不断完善“数据铁笼”，全力打造“制度铁笼”，严防严控重点工程和重点领域廉政风险，大力建设廉洁政府、效能政府，努力打造忠诚、干净、担当的公务员队伍。

　　各位代表，躬逢伟大的时代，我们充满无限的豪情和勇气；肩负伟大的事业，我们感到无比的自豪和荣光。时代潮流，浩浩荡荡，我们要做弄潮儿永立潮头；历史车轮，滚滚向前，我们要做奋斗者乘势而上。

　　潮平两岸阔，风正一帆悬。伟大的时代呼唤着我们，伟大的事业激励着我们。让我们紧密团结在以习近平同志为核心的党中央周围，鼓足勇气、卯足干劲，奋力创造出无愧于历史、无愧于时代、无愧于人民的辉煌业绩，大踏步奔向爽爽贵阳更加幸福美好的新未来！
